# Assessment of the knowledge, attitudes and practices level on toxoplasmosis and its seroprevalence in selected high-risk groups in Cebu, Philippines

**DOI:** 10.3389/fvets.2026.1758610

**Published:** 2026-07-24

**Authors:** Adrian P. Ybañez, Grahambell B. Sabanate, Serafin L. Garciano, Ruby H. Destajo, Rochelle Haidee D. Ybañez, Yoshifumi Nishikawa, Harvie P. Portugaliza

**Affiliations:** 1 College of Veterinary Medicine, Barili, Cebu, Philippines; 2 Institute for Molecular Genetics, Parasitology, and Vector-Borne Diseases at Main Campus, Cebu Technological University, Cebu, Philippines; 3 PhilVets Veterinary Products Trading, Cebu, Philippines; 4 National Research Center for Protozoan Diseases, Obihiro University of Agriculture and Veterinary Medicine, Obihiro, Hokkaido, Japan; 5 Faculty of Veterinary Medicine, Visayas State University, Baybay, Philippines

**Keywords:** Cebu, high-risk groups, knowledge-attitude-practice, Philippines, seroprevalence, *Toxoplasma gondii*

## Abstract

Toxoplasmosis, a zoonotic disease caused by *Toxoplasma gondii*, poses significant health risks, especially to high-risk individuals who are exposed to cats. This study aimed to assess the knowledge, attitudes, and practices (KAP) related to toxoplasmosis and determine its seroprevalence among selected high-risk groups in Cebu, Philippines—specifically veterinary students, veterinary personnel, and cat owners. A total of 416 participants completed a structured questionnaire evaluating their demographics, exposure history, and KAP levels, while 250 underwent further rapid serological testing for *T. gondii*. Results of the relative proportions revealed that there were more veterinary students who were having high level of knowledge (61%), compared to veterinary personnel (46%) and cat owners (24%). Relative proportions revealed that there were more veterinary students who were having high level of knowledge (61%), compared to veterinary personnel (46%) and cat owners (24%). Similar pattern was observed in the attitude level for veterinary students (54%), veterinary personnel (36%) and cat owners (32%). However, practice level showed that more veterinary personnel (54%) had high level than the veterinary students (35%) and cat owners (35%). The over-all seroprevalence of *T. gondii* was 29.2%, with the highest relative detection rates among veterinary personnel (43.8%) and cat owners (39%). While statistical analyses revealed no significant differences between the KAP levels of the three groups, significant associations between seropositivity and profile variables, including age, educational attainment, previous medical history, and frequency of cat contact (*P* ≤ 0.05) were found. These results highlight the need for sustained public education, improved veterinary and public health training, responsible pet ownership, and better access to diagnostic services.

## 1 Introduction

Toxoplasmosis, caused by the parasite *Toxoplasma gondii*, remains one of the most widespread protozoan diseases affecting animals and humans (1). It reportedly infects nearly one-third of the global human population (2–4). In the Philippines, the disease has been documented in animals (5, 6) and humans (6, 7). Cats reportedly had higher seropositivity rates than humans and pigs in Cebu, Philippines (6).

Despite its global prevalence, toxoplasmosis often remains understudied, particularly in regions with limited resources or competing public health priorities (8) or where congenital cases may be underestimated (including the Philippines) (9). The parasite was first detected in the Philippines in two hospital patients in 1950. Subsequent reports confirmed its presence in pigs, cats, and rats (6).

Its definitive hosts are the cats, which play a crucial role in its transmission by shedding infectious oocysts in their feces. In humans, it is detected using serological tests, which can indicate past or chronic infection (6). Humans can contract toxoplasmosis through various routes, including consuming undercooked meat containing tissue cysts, ingesting oocysts from contaminated food or water, or through mother-to-child transmission during pregnancy (10). While infections are typically asymptomatic in immunocompetent individuals, they can be severe or even fatal in immunocompromised patients and congenitally infected infants (35).

While the infection rates of Toxoplasmosis tend to vary (11), the disease is particularly concerning for high-risk groups, including veterinary personnel, veterinary students, and cat owners, as they frequently come into contact with the parasite through handling cats (12). Given their high-risk status, these groups must have a thorough understanding of toxoplasmosis, particularly its modes of transmission, prevention strategies, and control measures. Moreover, their knowledge, attitudes, and practices (KAP) related to toxoplasmosis is crucial for effective disease prevention and control (13).

As limited understanding about the disease may lead to misinformation (14), assessing the KAP among at-risk populations is crucial for identifying knowledge gaps and risky behaviors that may contribute to disease transmission and seroprevalence in the population, and for crafting effective prevention strategies. While studies in other countries have evaluated toxoplasmosis awareness among pregnant women (15), healthcare workers (36), and the general community (9), similar research in other at-risk groups in the Philippines is still limited. Veterinary personnel, veterinary students, and cat owners who frequently interact with cats are vulnerable to toxoplasmosis. However, their KAP and seroprevalence related to this disease remain understudied in the Philippines.

Populations with similar demographics in other countries have exhibited high seropositivity (16, 17), underscoring the zoonotic risk that may be linked to the expanding cat population. Therefore, assessing key risk factors is crucial to understand better the local dynamics (18).

## 2 Materials and methods

### 2.1 Research design and environment

This cross-sectional study employed a structured survey to assess respondents' toxoplasmosis KAP levels and serological testing using commercially available test kits to determine *T. gondii* seroprevalence among selected high-risk groups. The study was conducted in Cebu Island in Central Visayas (Region VII), Philippines, located at approximately 10°28′44.76″ N, 123°57′04.32″ E, with a land area of 4,943.72 km^2^ and a 2020 human census population of 3,325,385. This island is home to the second-largest economic capital city of the country, Cebu City, and the Philippines' richest province (Cebu). It is characterized by a mix of densely populated urban centers and rural/coastal communities where food handling, wet-market purchasing, backyard animal keeping, presence of stray cats, and frequent human–animal–environment contact may influence exposure opportunities relevant to toxoplasmosis. Cebu has documented evidence of endemic toxoplasmosis across key hosts, with reported antibody positivity of approximately 26.3% in humans, 42.3% in cats, and 13.4% in slaughtered pigs (6).

### 2.2 Research respondents

For the 2024–25 survey, 416 high-risk adult respondents participated. The high-risk groups were operationally defined as subpopulations with increased likelihood of exposure to *T. gondii* either through frequent occupational contact with animals/animal specimens or through regular household contact with cats and cat feces. These included veterinary students, veterinary personnel, and cat owners. Veterinary students were defined as currently enrolled veterinary medicine students with participation in animal handling and/or clinical–laboratory training activities. Veterinary personnel were defined as individuals currently working in veterinary-related settings (e.g., clinics, hospitals, laboratories, animal facilities in local government units) whose routine duties involved direct handling of animals and/or animal-derived specimens. To minimize heterogeneity, the cat-owner subgroup was defined as community-based adults who owned at least one cat and had regular household exposure, including personal responsibility for at least one cat-care activity (e.g., feeding and/or cleaning cat living areas/litter box maintenance).

From these sub-groups, 250 volunteers were serologically tested for toxoplasmosis. Inclusion criteria for all subgroups were age (≥18 years), residence in Cebu Province (at least 1 year), membership in one of the predefined high-risk subgroups as operationally defined, and provision of informed consent for the KAP survey and/or serologic testing. The exclusion criteria for the different sub-groups include non-enrolment for more than 1 semester for the veterinary students, non-exposure to animal contact for more than 6 months and separation from work for the veterinary personnel, and no contact with cats for at least 3 months for the cat owners. If the participant refuses to provide informed consent and/or fails to complete the questionnaire, they are also excluded from the study.

The study utilized purposive quota sampling to ensure adequate representation of the selected high-risk groups. Since participants were recruited non-randomly to meet predefined quotas, external validity may be limited, and the resulting sample may not accurately reflect the true distribution of characteristics, exposures, or toxoplasmosis risk profiles in the broader Cebu population or even within each target group. Consequently, the observed KAP patterns and seroprevalence estimates may be affected by selection bias and may not reflect provincial-level prevalence or attitudes. The findings should be interpreted as group-specific estimates within the recruited sample and are best viewed as exploratory evidence to inform targeted health education and hypothesis generation.

### 2.3 Research instruments

For the KAP survey, the questionnaire consisted of three sections. The majority of the questionnaire was adopted from Ybañez et al. (6) with slight modifications. This includes the demographic profile (section one) and KAP (section two). There were 10 statements for each of knowledge, attitude, and practice. The knowledge construct dealt with the understanding of toxoplasmosis, including transmission, symptoms, and health risks (6). The attitude construct included statements on personal beliefs and perceptions on risk, seriousness, and prevention of toxoplasmosis (18–25). The practices section contained statements relevant to behaviors on preventing infection, including hygiene practices and handling pets (6). The questionnaire was validated using Cronbach's alpha, which revealed an internal reliability coefficient of 0.81, indicating good reliability.

The statements were in four-point Likert scale to avoid neutral responses and central bias. Of the 30 statements, 14 were negatively stated to ensure that respondents read the different contents (26). Negatively stated statements were reverse-coded during data processing. Each response was assigned points (0 for I don't know/I can't remember, 1 for strongly disagree, 2 for disagree, 3 for agree, 4 for strongly agree). The total points per construct (KAP) served as the basis for categorizing levels per tercile (low, medium, high) with a minimum and maximum possible score of 0 and 40, respectively (27, 28).

For the blood collection, 3 ml syringes (with gauge 21 needles) and 2 ml plain tubes were used. Centrifugation was performed using standard centrifuge, while the serum was extracted using standard 200 ul micropipettes and transferred to 0.5 ml plain tubes before testing. For the serological tests, the collected serum was tested using a commercial rapid test kit (Toxo IgG/IgM test; Testsealabs, Hangzhou, China).

### 2.4 Research procedure

A letter request for participation was sent to identified veterinary hospital and clinics, veterinary department of local government units, veterinary students, and cat owners. After confirmation of participation, the survey form was provided online. Hardcopies were provided during an arranged schedule of visit when blood collection was conducted by a licensed medical technologist. After the survey, an infographic/brochure about toxoplasmosis were given to the respondents to raise awareness about toxoplasmosis. On the other hand, blood was processed for serum separation and storage at the Institute for Molecular Genetics, Parasitology, and Vector-borne Diseases (IMGPVD) at Cebu Technological University-Main Campus after each blood collection procedure.

Three milliliters of blood were aseptically collected by a licensed medical technologist following the guidelines of the World Health Organization (10). Collected blood was transferred to a plain red tube, and was subsequently centrifuged for 10 min. Serum was later collected and transferred to a 0.5 ml red microtainer and were labeled accordingly. Serum were stored at −20 °C until further use. The serum was tested using commercially available rapid test kit (Toxo IgG/IgM test; Testsealabs, Hangzhou, China) according to the manufacturer's instructions. This test kit has a reported 99.6% accuracy. Relative sensitivity and specificity for the Toxoplasma IgG were 92.6 and 99.0%, respectively. For the Toxoplasma IgM, Relative sensitivity and specificity were >99.9% and 99.5%, respectively.

Briefly, the pouch of the rapid test kit was first be brought to room temperature and then opened on a clean, level surface. Three drops of the serum sample (approximately 100 μl) was added to the specimen well, which was followed by two drops of buffer (70 μl). A positive result would show two lines: one in the control region (C) and another in the test line region (T). The formation of a burgundy-colored T2 band indicated a Toxoplasma IgG positive result, suggesting a recent or repeat infection, while a burgundy-colored T1 band indicated a Toxoplasma IgM positive result, suggesting a fresh infection. A negative result showed only a line in the control region (C).

The Toxoplasma IgG/IgM rapid test is suitable for disease screening. However, IgM reactivity is not a definitive indicator of recent infection, as anti-Toxoplasma IgM can persist for months to years, and some commercial assays may produce false-positive IgM results. This can lead to the misclassification of chronic infections as “acute” if confirmatory testing is not conducted. A single rapid test result cannot reliably determine the timing of infection, especially during pregnancy, because the timing of testing affects IgM detectability. Unfortunately, ELISA confirmatory test was not performed. The serologic findings in this study should primarily be interpreted as evidence of exposure (particularly via IgG) rather than a definitive classification of infection timing.

### 2.5 Data processing and analyses

Data from the filled-out hardcopy questionnaires were manually tabulated in a tally sheet and encoded into Microsoft Excel while data from the online survey form was later combined with the file. After data cleaning, the processed file was imported to using IBM SPSS Statistics for analysis. Descriptive statistics were employed. The seroprevalence of toxoplasmosis was determined using proportions. For univariate analysis, the significant associations between the profile variables, the KAP levels, and the *T. gondii* seropositivity of the respondents were evaluated using the Pearson chi-square test. Odds ratio was computed where applicable (in variable pairs that results in a 2x2 table). The significant differences of KAP levels between high-risk groups were evaluated using one-way ANOVA while signification association of KAP levels with the profile variables were evaluated using the Pearson chi-square test. Results were considered significant when *p* ≤ 0.05. For multivariate analyses, significant variables in the univariate analyses were evaluated using ordinal logistic regression or multinomial regression (if the test of parallel lines failed). Statistical significance was set at *p* ≤ 0.05. For the open-ended questions, emerging themes were identified. Responses were subsequently coded according to the emerging themes. Coded data were later processed using descriptive statistics.

### 2.6 Ethical considerations

The study obtained ethical clearance from the Local Research Ethics Committee of Cebu Technological University–Barili Campus (LREC Approval No. BA-2025-001). Its conduct adhered to the provisions of the Philippine Health Ethics Regulation Board and the Helsinki Declaration. Informed consent was sought from all respondents.

## 3 Results

### 3.1 Profile of respondents

A total of 416 high-risk respondents participated in the knowledge, attitude, and practice (KAP) survey. The respondents consisted of veterinary students, veterinary personnel, and cat owners. Most respondents were 18 to 25 years old, female, single, college-level, and from the medium socio-economic category. A high proportion of respondents owned cats, commonly with one to three cats, and most reported no present or previous medical conditions. Most respondents also reported frequent cat contact and ownership of animals other than cats, indicating relevant human-animal exposure contexts for toxoplasmosis risk assessment (Table 1).

**Table 1:** Profile of respondents at high risk for toxoplasma infection in Cebu, Philippines (*n* = 416).

Parameter	Veterinary students (*n* = 142)		Veterinary personnel (*n* = 103)		Cat owners (*n* = 171)		Total	
	Freq	%	Freq	%	Freq	%	Freq	%
Age (years)								
18–25	139	33.4	23	5.5	136	32.7	298	71.6
26–35	2	0.5	51	12.3	21	5	74	17.8
Above 35	1	0.2	29	7	14	3.4	44	10.6
Total	142	34.1	103	24.8	171	41.1	416	100
Biological sex at birth								
Female	110	26.4	58	13.9	129	31	297	71.4
Male	32	7.7	45	10.8	42	10.1	119	28.6
Total	142	34.1	103	24.8	171	41.1	416	100
Civil status								
Single	142	34.1	75	18	157	37.7	374	89.9
Married	—	—	26	6.3	14	3.4	40	9.6
Widowed	—	—	2	0.5	—	—	2	0.5
Total	142	34.1	103	24.8	171	41.1	416	100
Highest educational attainment								
College level	140	33.6	44	9.4	101	24.3	285	68.6
College graduate	2	0.5	59	14.2	70	16.8	131	31.5
Post graduate	0	0	4	1	0	0	0	0
ALS graduate	0	0	1	0.2	0	0	0	0
Total	142	34.1	103	24.8	171	41.1	416	100
Socio-economic status								
Low	44	10.6	34	8.2	38	9.1	116	27.9
Medium	97	23.3	68	16.3	129	31	294	70.7
High	1	0.2	1	0.2	4	1	6	1.4
Total	142	34.1	103	24.8	171	41.1	416	100
Present medical conditions								
No	138	33.2	91	21.9	163	39.2	392	94.2
Yes	4	1	12	2.9	8	1.9	24	5.8
Total	142	34.1	103	24.8	171	41.1	416	100
Previous medical conditions								
No	131	31.5	84	20.2	155	37.3	370	88.9
Yes	11	2.6	19	4.6	16	3.8	46	11.1
Total	142	34.1	103	24.8	171	41.1	416	100
Cat ownership								
No	66	15.9	56	13.5	—	—	122	29.3
Yes	76	18.3	47	11.3	171	41.1	294	70.7
Total	142	34.1	103	24.8	171	41.1	416	100
Number of cats								
None	66	15.9	56	13.5	—	—	122	29.3
1–3	43	10.3	31	7.5	119	28.6	193	46.4
4–6	25	6	11	2.6	37	8.9	73	17.5
7 or more	8	1.9	5	1.2	15	3.6	28	6.7
Total	142	34.1	103	24.8	171	41.1	416	100
Years of living with cats								
None	66	15.9	56	13.5	—	—	122	29.3
Less than 1	4	1	0	0	13	3.1	17	4.1
1–5	33	7.9	31	7.5	101	24.3	165	39.7
More than 5	39	9.4	16	3.8	57	13.7	112	26.9
Total	142	34.1	103	24.8	171	41.1	416	100
Frequency of contact with cats								
None	47	11.3	23	5.5	45	10.8	115	27.6
Less frequent	24	5.8	8	1.9	21	5	53	12.7
Frequent	71	17.1	72	17.3	105	25.2	248	59.6
Total	142	34.1	103	24.8	171	41.1	416	100
Ownership of other animals aside from cats								
No	25	6	35	8.4	62	14.9	122	29.3
Yes	117	28.1	68	16.3	109	26.2	294	70.7
Total	142	34.1	103	24.8	171	41.1	416	100
Consumption of street foods								
No	16	3.8	16	3.8	19	4.6	51	12.3
Yes	126	30.3	87	20.9	152	36.5	365	87.7
Total	142	34.1	103	24.8	171	41.1	416	100

### 3.2 Item-level knowledge, attitude, and practice responses on toxoplasmosis

**Figure 1:**
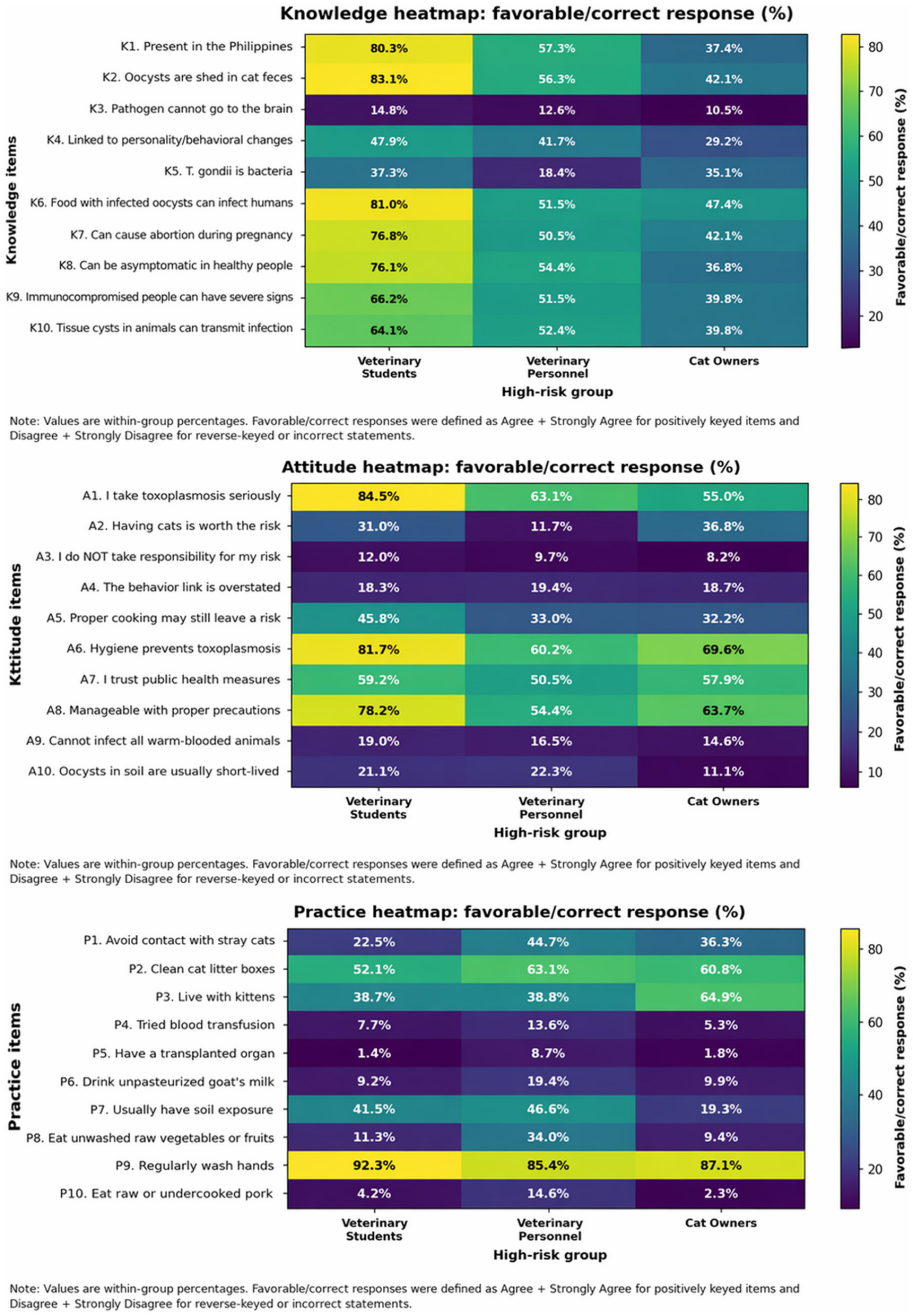
Item-level knowledge, attitude, and practice response patterns on toxoplasmosis among high-risk groups (*n* = 416).

The item-level heatmap showed clear differences in favorable or correct responses across the three high-risk groups. Veterinary students generally showed higher favorable or correct responses for knowledge items, particularly on the presence of toxoplasmosis in the Philippines, shedding of *Toxoplasma gondii* oocysts in cat feces, foodborne exposure, pregnancy-related risk, asymptomatic infection, and tissue cyst transmission. Veterinary personnel showed moderate item-level knowledge, while cat owners generally had lower favorable or correct responses across several transmission and disease-awareness items. These results indicate that formal veterinary training may improve recognition of toxoplasmosis risks, while household cat ownership alone may not ensure adequate disease knowledge (Figure 1; Supplementary Table S1).

For attitude items, veterinary students showed the highest favorable responses on taking toxoplasmosis seriously, confidence in hygiene as a preventive measure, and viewing toxoplasmosis as manageable with proper precautions. Cat owners also showed relatively high favorable responses for hygiene-related and manageability items, although favorable responses were lower for some reverse-keyed items that reflected risk perception and personal responsibility. Veterinary personnel showed moderate favorable attitudes, suggesting that occupational exposure does not necessarily translate into uniformly stronger attitude scores across all attitude indicators. These findings highlight the need to distinguish general agreement with prevention from deeper understanding of specific toxoplasmosis risks (Figure 1; Supplementary Table S1).

For practice items, favorable responses were highest for regular handwashing across all groups, especially among veterinary students and cat owners. However, favorable responses were lower for several exposure-related items, including avoidance of stray cats, unwashed raw vegetables or fruits, unpasteurized goat milk, raw or undercooked pork, soil exposure, and selected health-history indicators. These patterns suggest that protective hygiene behavior may coexist with exposure-related practices or conditions that can increase toxoplasmosis risk. This item-level pattern supports the need for practical, behavior-specific prevention messages rather than general awareness alone (Figure 1; Supplementary Table S1).

### 3.3 Distribution of KAP levels

**Figure 2:**
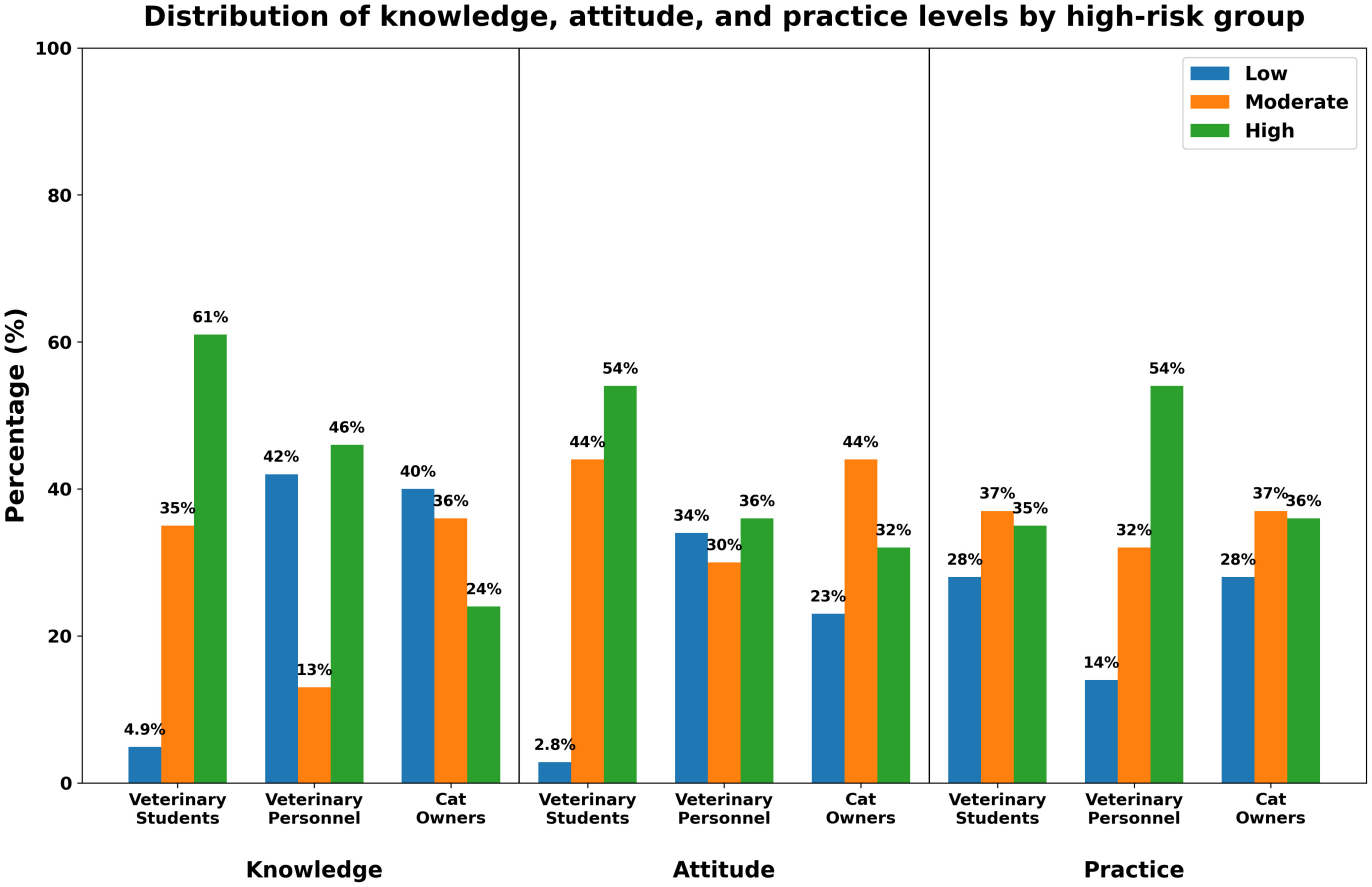
Distribution of knowledge, attitude, and practice levels on toxoplasmosis by high-risk group (*n* = 416). Percentages represent the proportion of respondents within each high-risk group.

The distribution of KAP levels showed that veterinary students had the highest proportion of respondents classified under the high knowledge level, followed by veterinary personnel and cat owners. A similar pattern was observed for attitude, with veterinary students having the highest proportion of high attitude level, while veterinary personnel and cat owners showed lower but comparable proportions. For practice, veterinary personnel had the highest proportion of respondents classified under the high level, while veterinary students and cat owners showed similar distributions across low, moderate, and high levels. These descriptive findings show that knowledge and attitude were strongest among veterinary students, whereas reported practice levels were strongest among veterinary personnel (Figure 2).

These descriptive patterns indicate that different high-risk groups may require different intervention priorities. Cat owners may require stronger foundational education, especially on transmission and food safety. Veterinary students may require stronger translation of knowledge into routine protective practice. Veterinary personnel may require continued reinforcement of updated zoonotic disease concepts and client-facing risk communication (Figure 2).

### 3.4 Serological detection of *Toxoplasma gondii*

**Figure 3:**
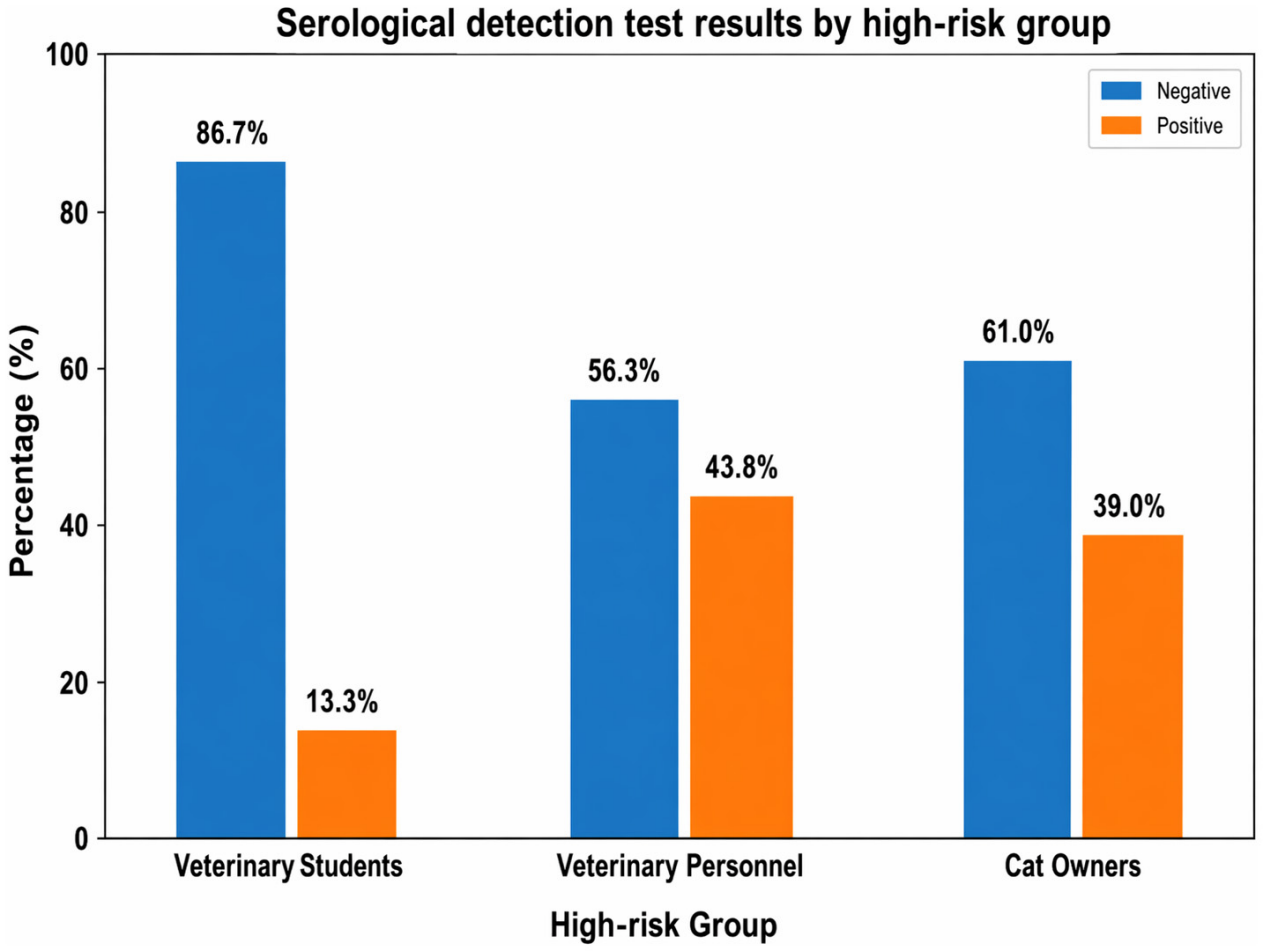
Serological detection test results for *Toxoplasma gondii* by high-risk group (*n* = 250). Percentages represent the proportion of respondents within each high-risk group.

Among the 250 respondents who underwent serological testing, 73 were positive for *T. gondii*, yielding an overall seropositivity of 29.2%. Veterinary personnel had the highest seropositivity, followed by cat owners and veterinary students. Veterinary students showed the lowest seropositivity and the highest proportion of negative results among the tested groups. The observed pattern suggests that occupational and household exposure contexts may contribute to differences in *T. gondii* exposure (Figure 3).

The higher seropositivity among veterinary personnel may reflect repeated occupational contact with animals, animal-derived materials, and potentially contaminated environments. The relatively high seropositivity among cat owners also supports the importance of household exposure and environmental hygiene in toxoplasmosis prevention. However, because serological testing was conducted only among 250 volunteers, the tested sample may not fully represent the complete survey sample. The findings should be interpreted as exposure estimates within the tested subgroup rather than provincial-level seroprevalence estimates (Figure 3).

### 3.5 Statistical associations of KAP levels and serological positivity

The statistical analyses showed significant associations between KAP levels and several respondent characteristics. Knowledge level was significantly associated with age, sex, civil status, high-risk group category, educational attainment, profession, socio-economic status, previous medical condition, nature and frequency of cat contact, ownership of other animals, and pregnancy status. Attitude level was significantly associated with age, sex, civil status, high-risk group category, educational attainment, profession, socio-economic status, current medical condition, constant cat contact, ownership of other animals, and pregnancy status. These findings suggest that knowledge and attitude were shaped by demographic, educational, occupational, and exposure-related factors (Table 2).

**Table 2:** Significant bivariate associations of KAP levels and serological positivity among high-risk respondents.

Parameters	Chi-square	df	*P*-value
Knowledge level with age category	28.532	4	<0.01
Knowledge level with sex	25.469	2	<0.01
Knowledge level with civil status	20.696	4	<0.01
Knowledge level with high-risk group category	80.924	4	<0.01
Knowledge level with highest educational attainment	51.680	4	<0.01
Knowledge level with type of profession	80.639	22	<0.01
Knowledge level with socio-economic level	11.716	4	0.020
Knowledge level with previous medical condition	11.055	2	<0.01
Knowledge level with constant contact with cats	12.998	2	<0.01
Knowledge level with frequency of contact with cats	14.459	4	<0.01
Knowledge level with ownership with other animals aside from cats	18.629	2	<0.01
Knowledge level with being pregnant	28.949	4	<0.01
Attitude level with age category	31.12	4	<0.01
Attitude level with biological sex	28.285	2	<0.01
Attitude level with civil status	25.797	4	<0.01
Attitude level with high-risk group category	55.246	4	<0.01
Attitude level with highest educational attainment	54.831	4	<0.01
Attitude level with type of profession	65.857	22	<0.01
Attitude level with socio-economic level	15.407	4	<0.01
Attitude level with present medical condition	6.819	2	0.033
Attitude level with constant contact with cats	7.357	2	0.025
Attitude level with ownership with other animals aside from cats	10.876	2	<0.01
Attitude level with being pregnant	27.925	4	<0.01
Practice level with high-risk group category	13.841	4	<0.01
Practice level with highest educational attainment	10.498	4	0.033
Practice level with present medical condition	6.576	2	0.037
Practice level with previous medical condition	7.901	2	0.019
Practice level with constant contact with cats	7.57	2	0.023
Serological positivity and age category	22.903	2	<0.01
Serological positivity and high-risk group category category	25.608	2	<0.01
Serological positivity and educational attainment	25.206	2	<0.01
Serological positivity and profession	27.436	9	<0.01
Serological positivity and frequency of cat contact	10.762	2	<0.01
Serological positivity and previous medical condition	4.236	1	0.040

KAP, knowledge, attitude, and practice.

Statistical significance was set at *p* ≤ 0.05.

Practice level showed fewer significant associations than knowledge and attitude. Significant associations were observed for high-risk group category, educational attainment, current medical condition, previous medical condition, and constant cat contact. This indicates that practice patterns may be less strongly shaped by general demographic variables than knowledge and attitude. Instead, practices may depend more on direct exposure, occupational routines, health-related factors, and specific behavioral contexts (Table 2).

Serological positivity was significantly associated with previous medical condition. Seropositivity was higher among respondents with a previous medical condition than among those without a previous medical condition. This association should be interpreted cautiously because the variable was broad and may represent heterogeneous clinical histories, exposure patterns, or residual confounding by age, occupation, and animal contact. The cross-sectional design also precludes causal interpretation (Table 2).

The one-way ANOVA showed significant differences in knowledge, attitude, and practice levels across high-risk groups. Veterinary students had significantly higher knowledge and attitude scores than veterinary personnel and cat owners, while veterinary personnel had significantly higher practice scores than veterinary students and cat owners. These results confirm that the differences observed descriptively were statistically supported and that KAP patterns varied by high-risk group and domain. The findings also indicate that stronger knowledge and attitudes do not automatically correspond to stronger reported practices (Table 2; Figure 2).

### 3.6 Emerging themes on main concerns about toxoplasmosis

**Figure 4:**
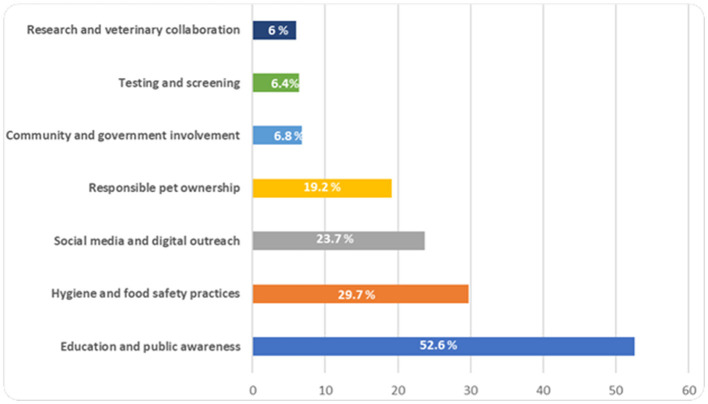
Emerging themes on main concerns about toxoplasmosis among respondents (*n* = 416). Percentages were computed from respondents who provided open-ended responses. Multiple themes per respondent were allowed; hence, percentages do not sum to 100%.

A total of 266 respondents provided open-ended responses on their main concerns about toxoplasmosis. The most frequently identified theme was education and public awareness, followed by hygiene and food safety practices, social media and digital outreach, and responsible pet ownership. Less frequent but still relevant themes included community and government involvement, testing and screening, and research and veterinary collaboration. Since multiple themes could be coded from each response, the percentages represent theme occurrence among respondents who provided qualitative responses and do not sum to 100% (Figure 4).

These qualitative findings indicate that respondents recognized the need for improved public education and clearer prevention-oriented messaging. The prominence of hygiene and food safety also aligns with the quantitative findings showing item-level gaps related to transmission and exposure-related practices. The themes support a prevention strategy that integrates public education, food safety, responsible pet ownership, accessible screening, and veterinary-public health collaboration. These results complement the quantitative findings and provide practical directions for community-based toxoplasmosis interventions (Figure 4).

## 4 Discussion

### 4.1 KAP patterns across high-risk groups

The item-level heatmap showed that veterinary students had the strongest favorable or correct responses for several knowledge items, particularly those related to local disease presence, oocyst shedding in cats, foodborne infection, pregnancy-related risk, asymptomatic infection, and tissue cyst transmission. This pattern is consistent with their formal academic exposure to parasitology, zoonoses, and public health concepts, which may strengthen recognition of transmission pathways and disease consequences (2, 6, 29). Veterinary personnel showed intermediate knowledge patterns, while cat owners showed lower favorable or correct responses across several knowledge items. These findings support the need for group-specific educational strategies, especially for cat owners who may experience frequent household exposure but have less formal access to zoonotic disease information (10, 30) (Figure 1).

Attitude patterns also varied across high-risk groups. Veterinary students showed stronger favorable attitudes toward seriousness, hygiene-based prevention, and manageability of toxoplasmosis, suggesting that formal training may influence risk appraisal and perceived responsibility (10, 31). Cat owners showed relatively favorable responses for hygiene and manageability but weaker responses for some reverse-keyed risk perception items, suggesting that general agreement with prevention may not consistently reflect deeper understanding of risk. Veterinary personnel showed moderate attitude scores, indicating that practical occupational experience may not be sufficient without continued professional education and reinforcement of zoonotic risk communication (32, 33) (Figure 1).

Practice responses showed a more complex pattern. Regular handwashing was highly endorsed across all groups, indicating broad awareness of basic hygiene as a protective behavior. However, lower favorable responses were observed for several exposure-related items, including dietary practices, soil exposure, stray cat contact, and other exposure-related indicators, suggesting that favorable hygiene practices may coexist with behaviors or circumstances that can sustain *T. gondii* exposure risk (10, 22). This supports the common KAP gap in which knowledge and attitudes do not always translate into consistently protective practices, especially when behaviors are shaped by routine, convenience, occupational exposure, or household habits (Figure 1).

### 4.2 Distribution of KAP levels

The KAP level distribution showed that veterinary students had the highest proportion of high knowledge and high attitude levels, while veterinary personnel had the highest proportion of high practice levels. This finding is important because it demonstrates a domain-specific pattern rather than a uniform advantage for one high-risk group. Veterinary students may benefit from structured coursework and recent exposure to theoretical concepts, while veterinary personnel may benefit from routine occupational practices that reinforce infection prevention behaviors (10, 31). Cat owners had lower knowledge levels than veterinary students and veterinary personnel, which indicates the need for accessible community-level education on toxoplasmosis transmission and prevention (Figure 2).

The ANOVA results further supported the descriptive findings by showing significant group differences in knowledge, attitude, and practice levels. Veterinary students had significantly higher knowledge and attitude scores than veterinary personnel and cat owners, while veterinary personnel had significantly higher practice scores than the other two groups. This pattern indicates that cognitive domains and behavioral domains may be shaped by different mechanisms, with formal education influencing knowledge and risk perception, and occupational routines influencing reported preventive practices. Hence, intervention programs should not assume that high knowledge automatically results in better practices or that occupational exposure automatically produces stronger disease-specific knowledge (Table 2; Figure 2).

### 4.3 Serological detection and exposure implications

The overall seropositivity of 29.2% indicates substantial exposure to *T. gondii* among the tested high-risk respondents. This value is close to the previously reported human seropositivity in Cebu, which supports the continued endemicity of toxoplasmosis in the local setting (6). Veterinary personnel showed the highest seropositivity, followed by cat owners and veterinary students, suggesting that occupational and household exposure contexts may contribute to differences in infection risk. Since cats are definitive hosts that shed environmentally resistant oocysts, frequent animal handling, litter contact, and contact with contaminated environments may increase exposure opportunities among veterinary personnel and cat owners (12, 29) (Figure 3).

The lower seropositivity among veterinary students may reflect younger age distribution, shorter cumulative exposure period, or greater recent exposure to formal prevention concepts. In contrast, veterinary personnel may have longer cumulative occupational exposure and more frequent contact with animal patients, clinical environments, and potentially contaminated materials. Cat owners may also experience repeated household-level exposure through cat care, litter handling, soil contamination, or food-related pathways. These interpretations require caution because serological testing was performed only among volunteers, and the tested sample may not fully represent the complete survey sample (Figure 3).

### 4.4 Factors associated with serological positivity

The statistical analyses identified previous medical condition as significantly associated with serological positivity. Respondents with a previous medical condition had higher seropositivity than those without a previous medical condition, suggesting a possible relationship between health history and *T. gondii* exposure or antibody positivity. This may reflect differences in cumulative exposure, health-seeking behavior, immune status, age structure, or other unmeasured factors associated with previous illness. However, because previous medical condition was broadly defined and the analysis was cross-sectional, this finding should be interpreted as an association rather than evidence of causality (Table 2).

The biological plausibility of this association depends on the type of previous medical condition, which was not disaggregated in the present analysis. Immunocompromised individuals are known to be more vulnerable to severe toxoplasmosis, but the presence of a previous medical condition does not necessarily indicate immunosuppression or greater susceptibility to infection (18, 34). Future studies should classify medical history into clinically meaningful categories and adjust for age, occupation, education, cat contact, dietary practices, soil exposure, and other relevant covariates. This would help determine whether medical history is an independent correlate of seropositivity or a proxy for other exposure-related variables (Table 2).

### 4.5 Factors associated with KAP levels

Knowledge and attitude levels were associated with a broader range of respondent characteristics than practice levels. This suggests that cognitive and perceptual domains may be more sensitive to demographic, educational, occupational, and exposure-related factors. Age, sex, civil status, high-risk group category, educational attainment, profession, socio-economic status, animal ownership, cat contact, and pregnancy status may influence the way respondents' access, understand, and interpret toxoplasmosis-related information. These findings are consistent with prior evidence showing that toxoplasmosis awareness and prevention-related attitudes can vary substantially by education, profession, and risk exposure context (20, 30, 32) (Table 2).

Practice level showed fewer significant associations, indicating that preventive behavior may be shaped by more specific contextual and behavioral constraints. Even when respondents know about toxoplasmosis or view it as serious, practices may still be influenced by household routines, occupational demands, availability of protective equipment, food habits, convenience, and perceived immediacy of risk. This pattern supports the need to move beyond awareness campaigns alone and incorporate behavior-focused interventions. Practical messages should emphasize safe litter handling, handwashing after cat or soil contact, proper washing of fruits and vegetables, avoidance of undercooked meat, and responsible pet management (10, 22, 33) (Table 2; Figure 1).

### 4.6 Emerging themes and intervention priorities

The emerging themes showed that education and public awareness were the most common concerns raised by respondents. This qualitative pattern reinforces the quantitative evidence that knowledge gaps remain, especially among cat owners and for selected transmission-related items. Respondents also emphasized hygiene and food safety practices, indicating recognition that toxoplasmosis prevention should not focus only on cats but also on foodborne and environmental exposure pathways. This is consistent with evidence that *T. gondii* can be transmitted through oocyst-contaminated food or water and through tissue cysts in raw or undercooked meat (2, 22) (Dubey et al., 2020) (Figure 4).

Social media and digital outreach emerged as another major theme, suggesting that respondents viewed online platforms as practical channels for increasing public awareness. This may be particularly relevant for younger, digitally active respondents and for community-level messaging that must compete with misinformation or incomplete knowledge. Responsible pet ownership also emerged as an important theme, reflecting recognition of the role of cats in the *T. gondii* life cycle and the importance of proper litter management, veterinary care, and environmental hygiene. These themes support a One Health-oriented intervention strategy integrating veterinary education, community health communication, food safety promotion, and responsible pet ownership messaging (10, 30, 33) (Figure 4).

### 4.7 Implications for public health and veterinary education

The combined quantitative and qualitative findings indicate that toxoplasmosis prevention in Cebu should be targeted according to group-specific needs. Cat owners require stronger foundational education on transmission, food safety, environmental exposure, and safe litter handling. Veterinary students may benefit from applied training that translates strong knowledge and attitudes into consistent preventive practice. Veterinary personnel may benefit from continuing professional education focused on updated toxoplasmosis concepts, workplace infection prevention, client counseling, and One Health communication (Figures 1, 2, 4).

The serological findings further support the need for accessible screening and risk communication among high-risk groups. Veterinary personnel and cat owners had higher seropositivity than veterinary students, which suggests that repeated occupational or household exposure may be relevant in local transmission dynamics. However, the use of a rapid test and the absence of confirmatory ELISA or avidity testing limit interpretation of infection timing. The serological results should be interpreted primarily as evidence of exposure rather than definitive classification of recent or acute infection (Figure 3).

## 5 Conclusion

The study showed domain-specific differences in toxoplasmosis-related KAP among selected high-risk groups in Cebu, Philippines, with veterinary students showing stronger knowledge and attitude levels and veterinary personnel showing stronger reported practice levels. Serological testing indicated substantial exposure to *T. gondii*, with the highest seropositivity observed among veterinary personnel and cat owners. Previous medical condition was significantly associated with serological positivity, although this finding should be interpreted cautiously because of the broad nature of the variable and the cross-sectional design. The quantitative and qualitative findings support targeted One Health interventions that strengthen public education, food safety, responsible pet ownership, occupational infection prevention, accessible screening, and veterinary-public health collaboration.

## Data Availability

Datasets are available on request: The raw data supporting the conclusions of this article will be made available by the authors, without undue reservation.
