# Supplementary material for: Assessment of the knowledge, attitudes and practices level on toxoplasmosis and its seroprevalence in selected high-risk groups in Cebu, Philippines
Source: Front Vet Sci. 2026 Jul 24;13:1758610. doi: 10.3389/fvets.2026.1758610 (PMC13449146; doi:10.3389/fvets.2026.1758610)
Supplement: Supplementary file 1 [file Table_1.docx]

**Supplementary Material**

**Supplementary Table S1. Knowledge, attitude, and practice responses regarding toxoplasmosis among high-risk groups in Cebu, Philippines (n = 416)**

Note: The full item-level response distribution is presented as supplementary material because the main manuscript summarizes these data visually in Figure 1.

| **Statements** | **High-Risk Group** | **I don't know/**  **I can't remember** | | **Strongly Disagree** | | **Disagree** | | **Agree** | | **Strongly Agree** | | **Total** | | |  |
| --- | --- | --- | --- | --- | --- | --- | --- | --- | --- | --- | --- | --- | --- | --- | --- |
|  |  | **freq** | **%** | **freq** | **%** | **freq** | **%** | **freq** | **%** | **freq** | **%** | | **n** | **%** | |
| 1. Toxoplasmosis, caused by *Toxoplasma gondii*, is present in the Philippines. | Veterinary Students | 6 | 1.4 | 3 | 0.7 | 19 | 4.6 | 49 | 11.8 | 65 | 15.6 | | 142 | 34.1 | |
|  | Veterinary Personnel | 43 | 10.3 | 0 | 0 | 1 | 0.2 | 29 | 7 | 30 | 7.2 | | 103 | 24.8 | |
|  | Cat Owners | 74 | 17.8 | 17 | 4.1 | 16 | 3.8 | 33 | 7.9 | 31 | 7.5 | | 171 | 41.1 | |
|  | **Total** | 123 | 29.6 | 20 | 4.8 | 36 | 8.7 | 111 | 26.7 | 126 | 30.3 | | 416 | 100 | |
| 2. *T. gondii* oocysts are shed in the feces of cats. | Veterinary Students | 7 | 1.7 | 4 | 1 | 13 | 3.1 | 45 | 10.8 | 73 | 17.5 | | 142 | 34.1 | |
|  | Veterinary Personnel | 40 | 9.6 | 1 | 0.2 | 4 | 1 | 32 | 7.7 | 26 | 6.3 | | 103 | 24.8 | |
|  | Cat Owners | 72 | 17.3 | 14 | 3.4 | 13 | 3.1 | 39 | 9.4 | 33 | 7.9 | | 171 | 41.1 | |
|  | **Total** | 119 | 28.6 | 19 | 4.6 | 30 | 7.2 | 116 | 27.9 | 132 | 31.7 | | 416 | 100 | |
| 3. *T. gondii* pathogen CANNOT go the brain. | Veterinary Students | 27 | 6.5 | 7 | 1.7 | 14 | 3.4 | 54 | 13 | 40 | 9.6 | | 142 | 34.1 | |
|  | Veterinary Personnel | 46 | 11.1 | 4 | 1 | 9 | 2.2 | 19 | 4.6 | 25 | 6 | | 103 | 24.8 | |
|  | Cat Owners | 84 | 20.2 | 2 | 0.5 | 16 | 3.8 | 31 | 7.5 | 38 | 9.1 | | 171 | 41.1 | |
|  | **Total** | 157 | 37.7 | 13 | 3.1 | 39 | 9.4 | 104 | 25 | 103 | 24.8 | | 416 | 100 | |
| 4. Toxoplasmosis is linked to personality or behavioral changes. | Veterinary Students | 17 | 4.1 | 13 | 3.1 | 44 | 10.6 | 37 | 8.9 | 31 | 7.5 | | 142 | 34.1 | |
|  | Veterinary Personnel | 48 | 11.5 | 2 | 0.5 | 10 | 2.4 | 31 | 7.5 | 12 | 2.9 | | 103 | 24.8 | |
|  | Cat Owners | 77 | 18.5 | 11 | 2.6 | 33 | 7.9 | 33 | 7.9 | 17 | 4.1 | | 171 | 41.1 | |
|  | **Total** | 142 | 34.1 | 26 | 6.3 | 87 | 20.9 | 101 | 24.3 | 60 | 14.4 | | 416 | 100 | |
| 5. *T. gondii* is bacteria. | Veterinary Students | 21 | 5 | 29 | 7 | 24 | 5.8 | 25 | 6 | 43 | 10.3 | | 142 | 34.1 | |
|  | Veterinary Personnel | 43 | 10.3 | 4 | 1 | 15 | 3.6 | 12 | 2.9 | 29 | 7 | | 103 | 24.8 | |
|  | Cat Owners | 64 | 15.4 | 25 | 6 | 35 | 8.4 | 21 | 5 | 26 | 6.3 | | 171 | 41.1 | |
|  | **Total** | 128 | 30.8 | 58 | 14 | 74 | 17.8 | 58 | 13.9 | 98 | 23.6 | | 416 | 100 | |
| 6. Humans can get *T. gondii* infection by unknowingly consuming food with infected oocysts. | Veterinary Students | 8 | 1.9 | 4 | 1 | 15 | 3.6 | 51 | 12.3 | 64 | 15.4 | | 142 | 34.1 | |
|  | Veterinary Personnel | 46 | 11.1 | 1 | 0.2 | 3 | 0.7 | 32 | 7.7 | 21 | 5 | | 103 | 24.8 | |
|  | Cat Owners | 58 | 13.9 | 13 | 3.1 | 19 | 4.6 | 42 | 10.1 | 39 | 9.4 | | 171 | 41.1 | |
|  | **Total** | 112 | 26.9 | 18 | 4.3 | 37 | 8.9 | 125 | 30 | 124 | 29.8 | | 416 | 100 | |
| 7. *T. gondii* can cause abortion in pregnant mothers if they are infected during pregnancy. | Veterinary Students | 12 | 2.9 | 6 | 1.4 | 15 | 3.6 | 45 | 10.8 | 64 | 15.4 | | 142 | 34.1 | |
|  | Veterinary Personnel | 43 | 10.3 | 0 | 0 | 8 | 1.9 | 21 | 5 | 31 | 7.5 | | 103 | 24.8 | |
|  | Cat Owners | 66 | 15.9 | 14 | 3.4 | 19 | 4.6 | 38 | 9.1 | 34 | 8.2 | | 171 | 41.1 | |
|  | **Total** | 121 | 29.1 | 20 | 4.8 | 42 | 10.1 | 104 | 25 | 129 | 31 | | 416 | 100 | |
| 8. Toxoplasmosis can be asymptomatic (no clinical signs) in immunocompetent or healthy individuals. | Veterinary Students | 8 | 1.9 | 7 | 1.7 | 19 | 4.6 | 50 | 12 | 58 | 13.9 | | 142 | 34.1 | |
|  | Veterinary Personnel | 44 | 10.6 | 1 | 0.2 | 2 | 0.5 | 26 | 6.3 | 30 | 7.2 | | 103 | 24.8 | |
|  | Cat Owners | 72 | 17.3 | 14 | 3.4 | 22 | 5.3 | 39 | 9.4 | 24 | 5.8 | | 171 | 41.1 | |
|  | **Total** | 124 | 29.8 | 22 | 5.3 | 43 | 10.3 | 115 | 27.6 | 112 | 26.9 | | 416 | 100 | |
| 9. People infected with *T. gondii* and have HIV, cancer or immunocompromised condition can have severe signs. | Veterinary Students | 14 | 3.4 | 9 | 2.2 | 25 | 6 | 45 | 10.8 | 49 | 11.8 | | 142 | 34.1 | |
|  | Veterinary Personnel | 48 | 11.5 | 0 | 0 | 2 | 0.5 | 22 | 5.3 | 31 | 7.5 | | 103 | 24.8 | |
|  | Cat Owners | 72 | 17.3 | 13 | 3.1 | 18 | 4.3 | 38 | 9.1 | 30 | 7.2 | | 171 | 41.1 | |
|  | **Total** | 134 | 32.2 | 22 | 5.3 | 45 | 10.8 | 105 | 25.2 | 110 | 26.4 | | 416 | 100 | |
| 10. *T. gondii* can form tissue cysts in animals, which can be a source of transmission when eaten. | Veterinary Students | 18 | 4.3 | 9 | 2.2 | 24 | 5.8 | 51 | 12.3 | 40 | 9.6 | | 142 | 34.1 | |
|  | Veterinary Personnel | 43 | 10.3 | 1 | 0.2 | 5 | 1.2 | 32 | 7.7 | 22 | 5.3 | | 103 | 24.8 | |
|  | Cat Owners | 68 | 16.3 | 15 | 3.6 | 20 | 4.8 | 39 | 9.4 | 29 | 7 | | 171 | 41.1 | |
|  | **Total** | 129 | 31 | 25 | 6 | 49 | 11.8 | 122 | 29.3 | 91 | 21.9 | | 416 | 100 | |
| 11. I take toxoplasmosis seriously. | Veterinary Students | 2 | 0.5 | 2 | 0.5 | 18 | 4.3 | 45 | 10.8 | 75 | 18 | | 142 | 34.1 | |
|  | Veterinary Personnel | 31 | 7.5 | 0 | 0 | 7 | 1.7 | 32 | 7.7 | 33 | 7.9 | | 103 | 24.8 | |
|  | Cat Owners | 41 | 9.9 | 13 | 3.1 | 23 | 5.5 | 44 | 10.6 | 50 | 12 | | 171 | 41.1 | |
|  | **Total** | 74 | 17.8 | 15 | 3.6 | 48 | 11.5 | 121 | 29.1 | 158 | 38 | | 416 | 100 | |
| 12. I believe that having cats is worth the risk of toxoplasmosis. | Veterinary Students | 5 | 1.2 | 12 | 2.9 | 32 | 7.7 | 43 | 10.3 | 50 | 12 | | 142 | 34.1 | |
|  | Veterinary Personnel | 33 | 7.9 | 4 | 1 | 8 | 1.9 | 33 | 7.9 | 25 | 6 | | 103 | 24.8 | |
|  | Cat Owners | 41 | 9.9 | 31 | 7.5 | 32 | 7.7 | 47 | 11.3 | 20 | 4.8 | | 171 | 41.1 | |
|  | **Total** | 79 | 19 | 47 | 11 | 72 | 17.3 | 123 | 29.6 | 95 | 22.8 | | 416 | 100 | |
| 13. I do NOT take responsibility for managing my risk of toxoplasmosis. | Veterinary Students | 25 | 6 | 5 | 1.2 | 12 | 2.9 | 36 | 8.7 | 64 | 15.4 | | 142 | 34.1 | |
|  | Veterinary Personnel | 36 | 8.7 | 4 | 1 | 6 | 1.4 | 28 | 6.7 | 29 | 7 | | 103 | 24.8 | |
|  | Cat Owners | 61 | 14.7 | 2 | 0.5 | 12 | 2.9 | 33 | 7.9 | 63 | 15.1 | | 171 | 41.1 | |
|  | **Total** | 122 | 29.3 | 11 | 2.6 | 30 | 7.2 | 97 | 23.3 | 156 | 37.5 | | 416 | 100 | |
| 14. I think the link between toxoplasmosis and behavior is overstated. | Veterinary Students | 19 | 4.6 | 7 | 1.7 | 19 | 4.6 | 62 | 14.9 | 35 | 8.4 | | 142 | 34.1 | |
|  | Veterinary Personnel | 41 | 9.9 | 5 | 1.2 | 15 | 3.6 | 30 | 7.2 | 12 | 2.9 | | 103 | 24.8 | |
|  | Cat Owners | 61 | 14.7 | 6 | 1.4 | 26 | 6.3 | 40 | 9.6 | 38 | 9.1 | | 171 | 41.1 | |
|  | **Total** | 121 | 29.1 | 18 | 4.3 | 60 | 14.4 | 132 | 31.7 | 85 | 20.4 | | 416 | 100 | |
| 15. I believe proper cooking may still leave a risk of having toxoplasmosis. | Veterinary Students | 6 | 1.4 | 12 | 2.9 | 53 | 12.7 | 44 | 10.6 | 27 | 6.5 | | 142 | 34.1 | |
|  | Veterinary Personnel | 38 | 9.1 | 9 | 2.2 | 25 | 6 | 23 | 5.5 | 8 | 1.9 | | 103 | 24.8 | |
|  | Cat Owners | 51 | 12.3 | 16 | 3.8 | 39 | 9.4 | 38 | 9.1 | 27 | 6.5 | | 171 | 41.1 | |
|  | **Total** | 95 | 22.8 | 37 | 8.9 | 117 | 28.1 | 105 | 25.2 | 62 | 14.9 | | 416 | 100 | |
| 16. I am confident hygiene prevents toxoplasmosis. | Veterinary Students | 2 | 0.5 | 8 | 1.9 | 16 | 3.8 | 48 | 11.5 | 68 | 16.3 | | 142 | 34.1 | |
|  | Veterinary Personnel | 31 | 7.5 | 5 | 1.2 | 5 | 1.2 | 36 | 8.7 | 26 | 6.3 | | 103 | 24.8 | |
|  | Cat Owners | 23 | 5.5 | 5 | 1.2 | 24 | 5.8 | 47 | 11.3 | 72 | 17.3 | | 171 | 41.1 | |
|  | **Total** | 56 | 13.5 | 18 | 4.3 | 45 | 10.8 | 131 | 31.5 | 166 | 39.9 | | 416 | 100 | |
| 17. I trust public health measures to handle toxoplasmosis. | Veterinary Students | 4 | 1 | 23 | 5.5 | 31 | 7.5 | 46 | 11.1 | 38 | 9.1 | | 142 | 34.1 | |
|  | Veterinary Personnel | 34 | 8.2 | 4 | 1 | 13 | 3.1 | 32 | 7.7 | 20 | 4.8 | | 103 | 24.8 | |
|  | Cat Owners | 25 | 6 | 17 | 4.1 | 30 | 7.2 | 58 | 13.9 | 41 | 9.9 | | 171 | 41.1 | |
|  | **Total** | 63 | 15.1 | 44 | 11 | 74 | 17.8 | 136 | 32.7 | 99 | 23.8 | | 416 | 100 | |
| 18. I view toxoplasmosis as a manageable concern with proper precautions. | Veterinary Students | 4 | 1 | 5 | 1.2 | 22 | 5.3 | 56 | 13.5 | 55 | 13.2 | | 142 | 34.1 | |
|  | Veterinary Personnel | 35 | 8.4 | 4 | 1 | 8 | 1.9 | 33 | 7.9 | 23 | 5.5 | | 103 | 24.8 | |
|  | Cat Owners | 33 | 7.9 | 4 | 1 | 25 | 6 | 55 | 13.2 | 54 | 13 | | 171 | 41.1 | |
|  | **Total** | 72 | 17.3 | 13 | 3.1 | 55 | 13.2 | 144 | 34.6 | 132 | 31.7 | | 416 | 100 | |
| 19. I believe *T. gondii* CANNOT infect all warm-blooded animals. | Veterinary Students | 32 | 7.7 | 11 | 2.6 | 16 | 3.8 | 41 | 9.9 | 42 | 10.1 | | 142 | 34.1 | |
|  | Veterinary Personnel | 45 | 10.8 | 5 | 1.2 | 12 | 2.9 | 21 | 5 | 20 | 4.8 | | 103 | 24.8 | |
|  | Cat Owners | 75 | 18 | 5 | 1.2 | 20 | 4.8 | 38 | 9.1 | 33 | 7.9 | | 171 | 41.1 | |
|  | **Total** | 152 | 36.5 | 21 | 5 | 48 | 11.5 | 100 | 24 | 95 | 22.8 | | 416 | 100 | |
| 20. I believe *T. gondii* oocysts in the soil are usually short-lived. | Veterinary Students | 27 | 6.5 | 8 | 1.9 | 22 | 5.3 | 54 | 13 | 31 | 7.5 | | 142 | 34.1 | |
|  | Veterinary Personnel | 48 | 11.5 | 5 | 1.2 | 18 | 4.3 | 18 | 4.3 | 14 | 3.4 | | 103 | 24.8 | |
|  | Cat Owners | 83 | 20 | 4 | 1 | 15 | 3.6 | 43 | 10.3 | 26 | 6.3 | | 171 | 41.1 | |
|  | **Total** | 158 | 38 | 17 | 4.1 | 55 | 13.2 | 115 | 27.6 | 71 | 17.1 | | 416 | 100 | |
| 21. I avoid contact with stray cats. | Veterinary Students | 18 | 4.3 | 46 | 11 | 46 | 11.1 | 19 | 4.6 | 13 | 3.1 | | 142 | 34.1 | |
|  | Veterinary Personnel | 9 | 2.2 | 22 | 5.3 | 26 | 6.3 | 38 | 9.1 | 8 | 1.9 | | 103 | 24.8 | |
|  | Cat Owners | 25 | 6 | 32 | 7.7 | 52 | 12.5 | 37 | 8.9 | 25 | 6 | | 171 | 41.1 | |
|  | **Total** | 52 | 12.5 | 100 | 24 | 124 | 29.8 | 94 | 22.6 | 46 | 11.1 | | 416 | 100 | |
| 22. I clean cat litter boxes | Veterinary Students | 28 | 6.7 | 20 | 4.8 | 20 | 4.8 | 27 | 6.5 | 47 | 11.3 | | 142 | 34.1 | |
|  | Veterinary Personnel | 13 | 3.1 | 9 | 2.2 | 16 | 3.8 | 44 | 10.6 | 21 | 5 | | 103 | 24.8 | |
|  | Cat Owners | 9 | 2.2 | 24 | 5.8 | 34 | 8.2 | 40 | 9.6 | 64 | 15.4 | | 171 | 41.1 | |
|  | **Total** | 50 | 12 | 53 | 13 | 70 | 16.8 | 111 | 26.7 | 132 | 31.7 | | 416 | 100 | |
| 23. I live with kittens. | Veterinary Students | 37 | 8.9 | 35 | 8.4 | 20 | 4.8 | 18 | 4.3 | 32 | 7.7 | | 142 | 34.1 | |
|  | Veterinary Personnel | 14 | 3.4 | 12 | 2.9 | 28 | 6.7 | 28 | 6.7 | 21 | 5 | | 103 | 24.8 | |
|  | Cat Owners | 14 | 3.4 | 67 | 16 | 44 | 10.6 | 17 | 4.1 | 29 | 7 | | 171 | 41.1 | |
|  | **Total** | 65 | 15.6 | 114 | 27 | 92 | 22.1 | 63 | 15.1 | 82 | 19.7 | | 416 | 100 | |
| 24. I have tried blood transfusion. | Veterinary Students | 66 | 15.9 | 6 | 1.4 | 5 | 1.2 | 19 | 4.6 | 46 | 11.1 | | 142 | 34.1 | |
|  | Veterinary Personnel | 18 | 4.3 | 7 | 1.7 | 7 | 1.7 | 32 | 7.7 | 39 | 9.4 | | 103 | 24.8 | |
|  | Cat Owners | 87 | 20.9 | 5 | 1.2 | 4 | 1 | 16 | 3.8 | 59 | 14.2 | | 171 | 41.1 | |
|  | **Total** | 171 | 41.1 | 18 | 4.3 | 16 | 3.8 | 67 | 16.1 | 144 | 34.6 | | 416 | 100 | |
| 25. I have a transplanted organ. | Veterinary Students | 82 | 19.7 | 2 | 0.5 | 0 | 0 | 11 | 2.6 | 47 | 11.3 | | 142 | 34.1 | |
|  | Veterinary Personnel | 22 | 5.3 | 4 | 1 | 5 | 1.2 | 28 | 6.7 | 44 | 10.6 | | 103 | 24.8 | |
|  | Cat Owners | 99 | 23.8 | 1 | 0.2 | 2 | 0.5 | 8 | 1.9 | 61 | 14.7 | | 171 | 41.1 | |
|  | **Total** | 203 | 48.8 | 7 | 1.7 | 7 | 1.7 | 47 | 11.3 | 152 | 36.5 | | 416 | 100 | |
| 26. I drink fresh (unpasteurized) goat's milk. | Veterinary Students | 66 | 15.9 | 6 | 1.4 | 7 | 1.7 | 19 | 4.6 | 44 | 10.6 | | 142 | 34.1 | |
|  | Veterinary Personnel | 15 | 3.6 | 4 | 1 | 16 | 3.8 | 27 | 6.5 | 41 | 9.9 | | 103 | 24.8 | |
|  | Cat Owners | 83 | 20 | 7 | 1.7 | 10 | 2.4 | 14 | 3.4 | 57 | 13.7 | | 171 | 41.1 | |
|  | **Total** | 164 | 39.4 | 17 | 4.1 | 33 | 7.9 | 60 | 14.4 | 142 | 34.1 | | 416 | 100 | |
| 27. I engage in activities that usually has contact or exposure to soil. | Veterinary Students | 24 | 5.8 | 24 | 5.8 | 35 | 8.4 | 33 | 7.9 | 26 | 6.3 | | 142 | 34.1 | |
|  | Veterinary Personnel | 14 | 3.4 | 16 | 3.8 | 32 | 7.7 | 23 | 5.5 | 18 | 4.3 | | 103 | 24.8 | |
|  | Cat Owners | 45 | 10.8 | 12 | 2.9 | 21 | 5 | 46 | 11.1 | 47 | 11.3 | | 171 | 41.1 | |
|  | **Total** | 83 | 20 | 52 | 13 | 88 | 21.2 | 102 | 24.5 | 91 | 21.9 | | 416 | 100 | |
| 28. I eat unwashed raw vegetables or fruits. | Veterinary Students | 44 | 10.6 | 3 | 0.7 | 13 | 3.1 | 32 | 7.7 | 50 | 12 | | 142 | 34.1 | |
|  | Veterinary Personnel | 13 | 3.1 | 6 | 1.4 | 29 | 7 | 23 | 5.5 | 32 | 7.7 | | 103 | 24.8 | |
|  | Cat Owners | 70 | 16.8 | 4 | 1 | 12 | 2.9 | 13 | 3.1 | 72 | 17.3 | | 171 | 41.1 | |
|  | **Total** | 127 | 30.5 | 13 | 3.1 | 54 | 13 | 68 | 16.3 | 154 | 37 | | 416 | 100 | |
| 29. I regularly wash my hands. | Veterinary Students | 0 | 0 | 3 | 0.7 | 8 | 1.9 | 48 | 11.5 | 83 | 20 | | 142 | 34.1 | |
|  | Veterinary Personnel | 6 | 1.4 | 1 | 0.2 | 8 | 1.9 | 46 | 11.1 | 42 | 10.1 | | 103 | 24.8 | |
|  | Cat Owners | 3 | 0.7 | 6 | 1.4 | 13 | 3.1 | 37 | 8.9 | 112 | 26.9 | | 171 | 41.1 | |
|  | **Total** | 9 | 2.2 | 10 | 2.4 | 29 | 7 | 131 | 31.5 | 237 | 57 | | 416 | 100 | |
| 30. I eat raw or undercooked pork meat. | Veterinary Students | 63 | 15.1 | 0 | 0 | 6 | 1.4 | 18 | 4.3 | 55 | 13.2 | | 142 | 34.1 | |
|  | Veterinary Personnel | 13 | 3.1 | 4 | 1 | 11 | 2.6 | 27 | 6.5 | 48 | 11.5 | | 103 | 24.8 | |
|  | Cat Owners | 76 | 18.3 | 1 | 0.2 | 3 | 0.7 | 17 | 4.1 | 74 | 17.8 | | 171 | 41.1 | |
|  | **Total** | 152 | 36.5 | 5 | 1.2 | 20 | 4.8 | 62 | 14.9 | 177 | 42.5 | | 416 | 100 | |
